# The dynamics of the antibiotic resistome in the feces of freshly weaned pigs following therapeutic administration of oxytetracycline

**DOI:** 10.1038/s41598-019-40496-8

**Published:** 2019-03-11

**Authors:** Mahdi Ghanbari, Viviana Klose, Fiona Crispie, Paul D. Cotter

**Affiliations:** 1 BIOMIN Research Center, Tulln, Austria; 2 Teagasc Food Research Centre, Moorepark, Fermoy, Cork, and APC Microbiome Ireland, Cork, Ireland

## Abstract

In this study, shotgun metagenomics was employed to monitor the effect of oxytetracycline, administered at a therapeutic dose, on the dynamics of the microbiota and resistome in the feces of weaned pigs. Sixteen weaning pigs were assigned to one of two treatments including standard starter diet for 21 days or antibiotic-supplemented diet (10 g oxytetracycline/100 kg body weight/day) for 7 days, followed by 14 days of standard starter diet. Feces were collected from the pigs on days 0, 8, and 21 for microbiota and resistome profiling. Pigs receiving oxytetracycline exhibited a significantly greater richness (ANOVA, P = 0.034) and diversity (ANOVA, P = 0.048) of antibiotic resistance genes (ARGs) than the control pigs. Antibiotic administration significantly enriched the abundances of 41 ARGs, mainly from the tetracycline, betalactam and multidrug resistance classes. Compositional shifts in the bacterial communities were observed following 7 days of antibiotic adminstration, with the medicated pigs showing an increase in *Escherichia* (Proteobacteria) and *Prevotella* (Bacteroidetes) populations compared with the nonmedicated pigs. This might be explained by the potential of these taxa to carry ARGs that may be transferred to other susceptible bacteria in the densely populated gut environment. These findings will help in the optimization of therapeutic schemes involving antibiotic usage in swine production.

## Introduction

Antibiotics have been used for decades in swine and other livestock production for both therapeutic (e.g. treatment of specific diseases) and nontherapeutic (growth promotion) purposes^1,2^. For years, nontherapeutic (low-dose) application of antibiotics as growth promoters was linked with beneficial effects; however, there is data that supports the fact that this practice possible contributes to the emergence of antimicrobial-resistant bacteria, thus exacerbating the problem of antibiotic resistance in animal and human pathogens^2–10^. Additionally, it is likely that therapeutic doses of antibiotics can be subinhibitory antibiotic concentrations for some host-associated bacteria, enhancing the selection for antibiotic resistance genes (ARGs) and the horizontal transfer of these genes^4^.

Tetracyclines are a broad spectrum and relatively low cost group of antibiotics, of which tetracycline, chlortetracycline and oxytetracycline are frequently employed in veterinary medicine^5,11,12^. Tetracyclines have several therapeutic indications, which are associated with various infections in food-producing animals (e.g., infections caused by *Mycoplasma*, *Chlamydia*, *Pasteurella*, *Clostridium*, *Ornithobacterium rhinotracheale*, and some protozoa^11,12^). In food-producing species, including swine, first-generation tetracyclines (e.g., oxytetracycline) are most frequently employed. Therapeutic indications in animals comprise respiratory infections, dermal and soft tissue infections, peritonitis, metritis, and other enteric infections^5,8,11^. The recommended dosage of oxytetracycline for pigs for therapeutic purposes is 40 mg oxytetracycline hydrochloride/kg body weight (KBW)/day, for 7–10 days. In numerous countries, tetracyclines are included in feed, not just for therapeutic purposes but also at subtherapeutic doses to promote growth in swine^2,3,5,11,13^. Consumer apprehension associated with emerging bacterial resistance has led to antibiotics being no longer used for such purposes in some regions including the EU, the USA, New Zealand, Chile, Bangladesh, South Korea, and Vietnam^14,15^. However, nontherapeutic administration of tetracyclines for growth promotion purposes is still allowed in many other countries^3,11,12^.

We hypothesized that a therapeutic application of antibiotic could cause detectable and long term changes on pig fecal microbiota and resistome composition. To test this theory, we investigated the effect of a 7-day oxytetracycline administration at therapeutic dose and its withdrawal, on the diversity and abundance of the fecal antibiotic resistome and microbiota in freshly weaned pigs using a whole-metagenome shotgun sequencing approach. The findings of this study have important implications for swine production and public health, since there is a paucity of research on the effects of therapeutic doses of antibiotics on the diversity and abundance of the gut microbiota as well as the antibiotic resistome.

## Results

Within the first week of the feeding trial, three pigs (two from the antibiotic-medicated group and one from the control group) showed symptoms of *E. coli* infection and were treated once with the anti-inflammatory drug dexamethasone as well as with fluoroquinolone (3rd generation, 3 mL/100 KBW) for three consecutive days. Therefore, metagenome data from these animals were not included in the downstream data processing.

### Sequencing

Sequencing generated approximately 600 million sequences, ranging from 8.18 to 19 million per sample (Supplementary Table S1). The average quality score (Phred scores) across all the samples was 35.11 and ranged from 32.6 to 40. Phred scores greater than Q30 indicated that there was a less than 0.1% chance that a base was called incorrectly. Quality filtering of datasets resulted in the removal of 0.19% of the reads with Phred score <33 as well as removal of 2.5% of the reads that were classified as belonging to the host and PhiX genome.

### Resistome diversity and composition of the gut

**Figure 1:**
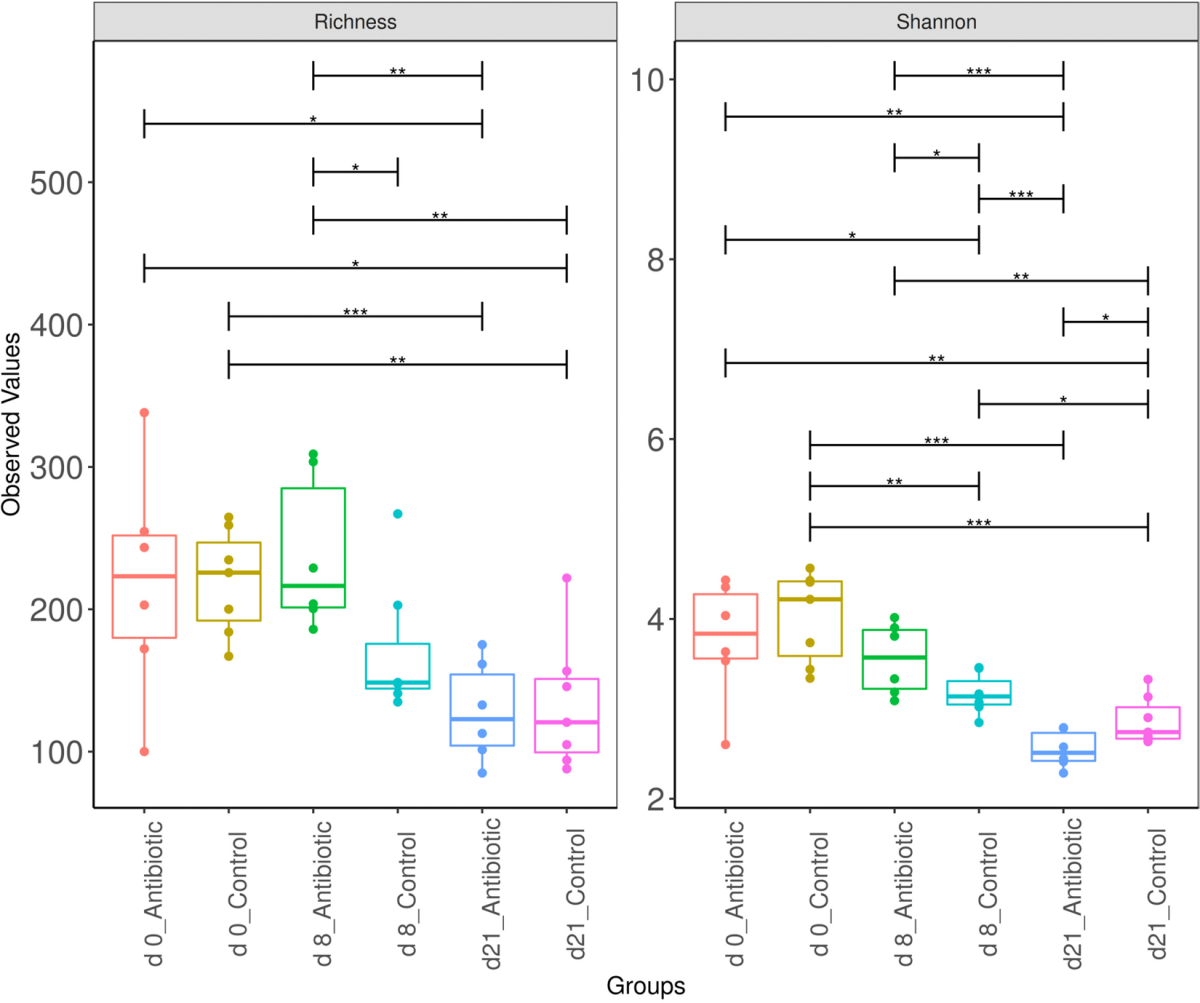
Richness and diversity (Shannon) of antibiotic resistance genes across treatments and time points. The richness and diversity are presented with the median values indicated (central black horizontal lines); the 25th and 75th percentiles are indicated (boxes), and the whiskers extend from each end of the box to the most extreme values within 1.5 times the interquartile range from the respective end. ANOVA, *P < 0.05, **P < 0.01, and ***P < 0.001, respectively.

Using the MEGARes database with a 90% gene cutoff fraction, 490,000 reads were aligned to 648 AMR genes across both groups. The AMR genes were classified into 19 unique classes of resistance, 49 mechanisms and 175 groups (Supplementary Tables S2 and S3). Following antibiotic administration for 7 days, the pigs receiving oxytetracycline were enriched in ARGs and had high diversity of ARGs (Fig. 1). Alpha diversity analysis revealed that the overall size of the resistome (i.e., the number of unique ARGs) was significantly affected (ANOVA, P < 0.05) by the day as well as by the oxytetracycline treatment (Fig. 1), with the highest resistome diversity in both groups observed on day 0 and the lowest diversity observed at the end of the trial. Linear mixed-effect analysis of the diversity indices showed a significant differences in the richness (P = 0.034) and diversity (P = 0.048, Shannon index) of ARGs between the pigs of the control and antibiotic-medicated groups from day 0 to day 8 but not at day 21 following the withdrawal period.

**Figure 2:**
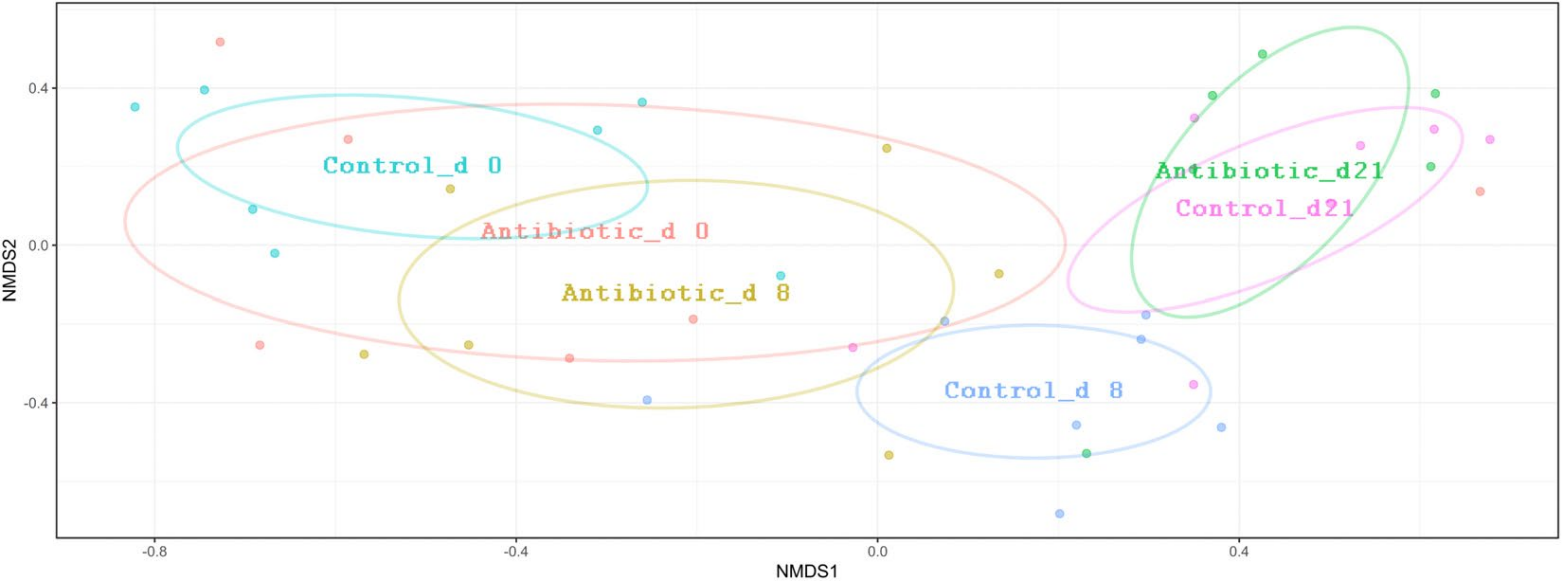
NMDS ordinations based on Bray–Curtis dissimilarity metric showing the changes in ARG compositions in antibiotic and control groups over time (stress = 0.095, R = 0.40, and P = 0.001). The low 2D stress values indicating that these data were well-represented by the two-dimensional ordinations. Ellipses indicate 95% confidence intervals of multivariate t-distribution around centroids of the groupings with treatment and sampling time points as factor.

In a further assessment of the resistome composition and diversity level, NMDS analysis based on the Bray-Curtis dissimilarity metric displayed a clear separation of the medicated animals from the nonmedicated animals samples at day 8 and 21 (Fig. 2). The two-way PerMANOVA test followed by pairwise post-hoc comparisons (https://github.com/leffj/mctoolsr/) showed a significant difference in the profile of the relative abundance of ARGs between the treatments for both day 8 (q = 0.038) and day 21 (q = 0.010) but not for day 0 (q = 0.262). While the medicated pigs medicated with oxytetracycline clearly diverged from the nonmedicated pigs at day 8 according to the NMDS ordination, the ARGs profile in the medicated group at day 21 tended to be closer to the nonmedicated group, indicating a resilience of the bacterial communities carrying ARGs from antibiotic perturbation.

**Figure 3:**
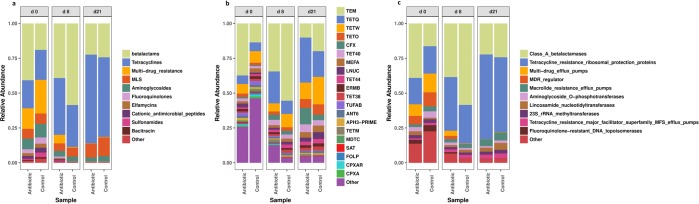
Normalized relative abundances of the top 10 classes (**a**) and mechanisms (**b**) as well as of the top 20 groups of ARGs (**c**) in the feces of medicated and nonmedicated pigs at different time points (d0, d8, d21).

Tetracycline resistance was the predominant class to which the reads were aligned, with beta-lactam resistance constituting most of the remaining reads (Fig. 3a). In fact, regardless of antibiotic medication, all the samples harbored a diverse range of ARGs (Fig. 3). In the tetracycline class, the main mechanism of resistance detected was through resistance ribosomal protection proteins (RRPPs). The main mechanism of resistance within the beta-lactam class was Class A beta-lactamases (CABLs). In addition to CABLs and RRPPs, the other predominant mechanisms of resistance were multidrug efflux pumps, multidrug resistance regulators, macrolide resistance efflux pumps, and lincosamide nucleotidyltransferases (Fig. 3b).

**Figure 4:**
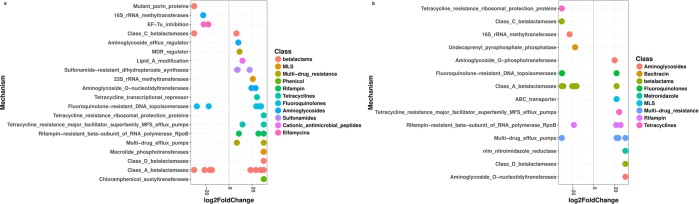
Significant (q < 0.05) log-fold changes in the abundances of ARG hits (summarized according to resistance mechanism and colored according to the class of resistance) in samples from day 0 to day 8 (**a**) and day 21 (**b**). Positive log-fold change point out an increase in abundance, while negative log-fold change point out a reduction in abundance over time in the antibiotic medicated pigs compared to the control group.

Differential abundance analysis revealed that from day 0 to day 8 (the last day of antibiotic administration), oxytetracycline feeding significantly enriched the abundances of 41 ARGs (q < 0.05), which were mainly from the tetracycline, beta-lactam and multidrug resistance classes (Fig. 4a, Supplementary Table S4). Further analysis of the samples at day 21, two weeks after the withdrawal of antibiotic administration, showed that 17 ARGs remained significantly more abundant (q < 0.05) in antibiotic-treated pigs than in the control group (Fig. 4b, Supplementary Table S5).

### Microbiome diversity and gut composition

**Figure 5:**
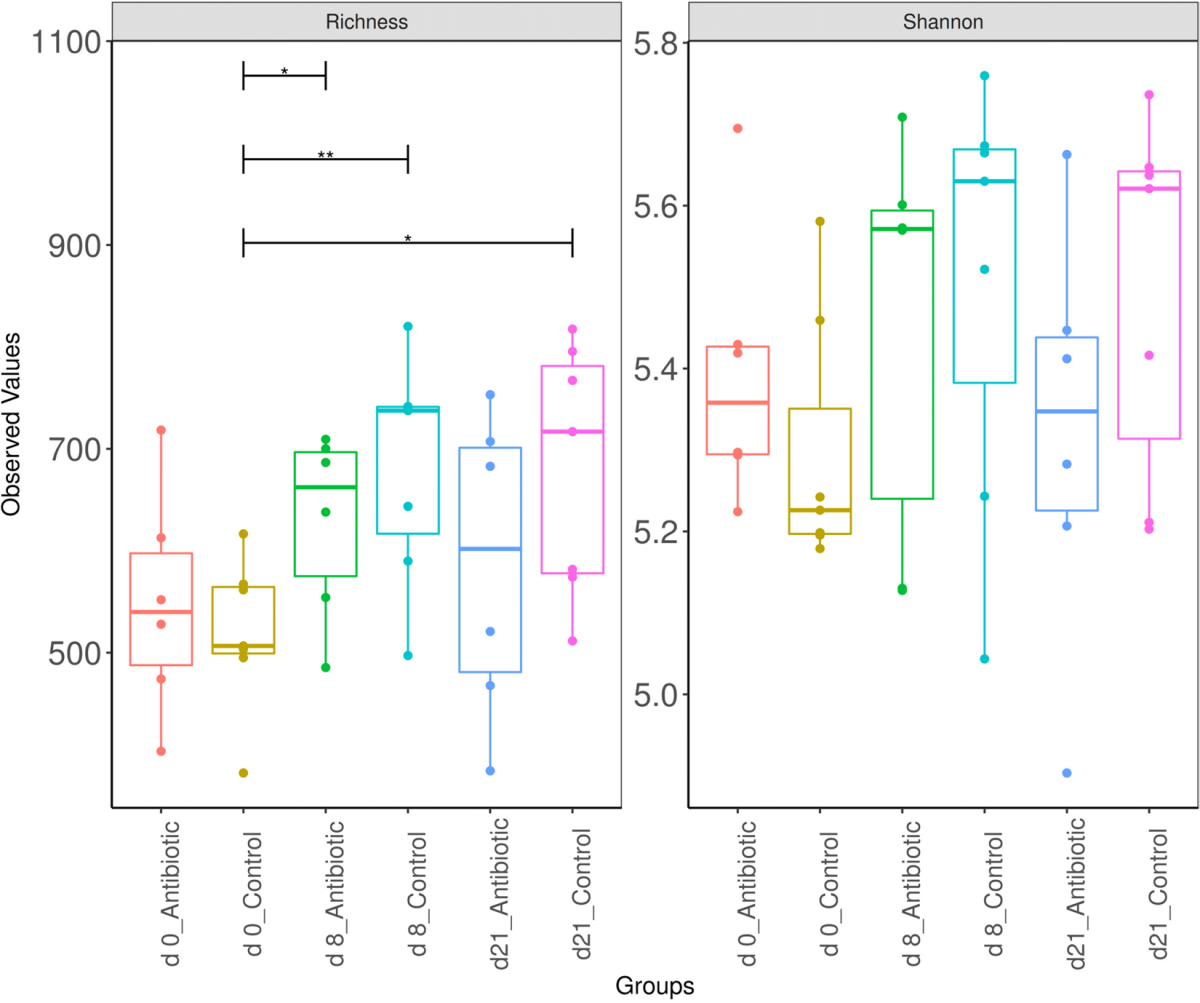
Richness and diversity (Shannon) of taxa across treatments and over time. The indices are presented with the median values indicated (central black horizontal lines); the 25th and 75th percentiles are indicated (boxes), and the whiskers extend from each end of the box to the most extreme values within 1.5 times the interquartile range from the respective end. Whiskers data points beyond this range are displayed as small black circles. ANOVA, *P < 0.05 and **P < 0.011, respectively.

Comparison of microbial community structure using the alpha diversity indices revealed that the total number of detected species (richness) as well as their diversity (Shannon) were lower for the communities in medicated animals than for the control animals during the antibiotic treatment period (day 0 to day 8) (Fig. 5), with the values decreasing further during the withdrawal period (Fig. 5).

**Figure 6:**
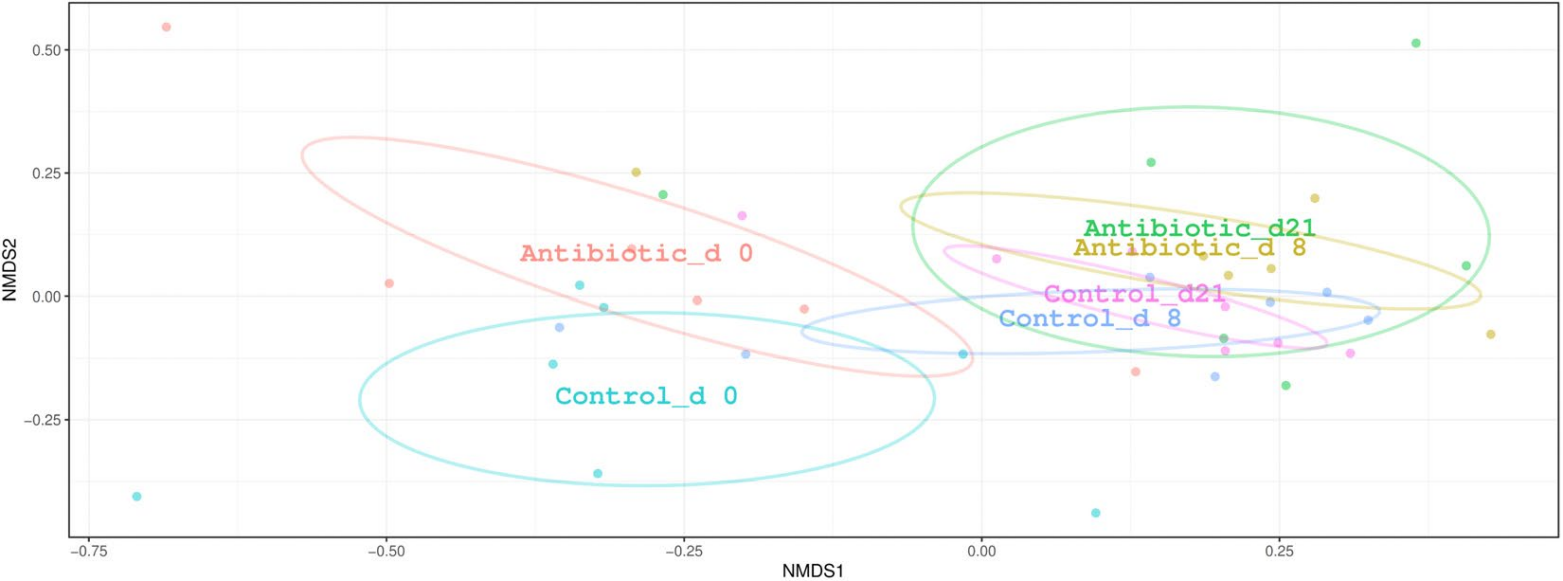
NMDS ordinations based on the Bray–Curtis dissimilarity metric showing the shift in the composition of bacterial community in antibiotic and control groups over time (stress = 0.14, R = 0.45, and P = 0.001. The 2D stress values was lower than 0.17 indicating that these data were well-represented by the two-dimensional ordinations. Ellipses indicate 95% confidence intervals of multivariate t-distribution around centroids of the groupings with treatment and sampling time points as factor. Each point represents a pig with sequences clustered based on classification at the species level.

The temporal shifts of bacterial communities were relatively similar to those of ARGs, with the medicated pigs diverged from the nonmedicated pigs (Fig. 6). In fact, analysis of the community structures showed significant differences between medicated and nonmedicated animals after antibiotic treatment (day 8, post hoc two-way PerMANOVA, q = 0.04). Taken together, these data indicate that oxytetracycline administration reduced both the bacterial community’ richness and diversity in the gut microbiota of the pigs and that the gut bacterial community diversity did not fully recover, despite the withdrawal of the antibiotic for two weeks.

**Figure 7:**
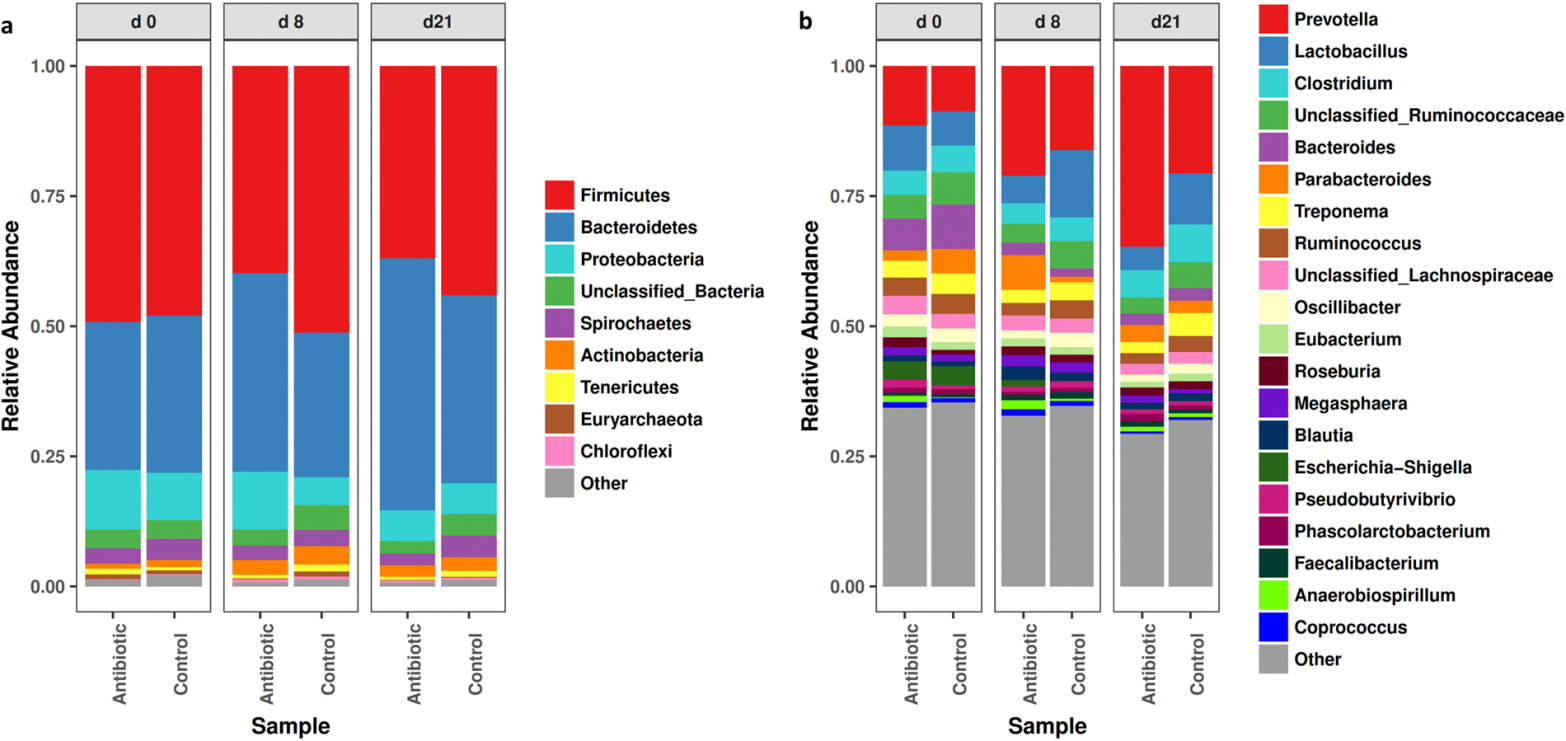
The 10 and 20 most abundant taxa in the bacterial communities at the phylum (**a**) and genus (**b**) levels, respectively, according to Metaxa2 analysis.

Taxonomic profiling of the fecal samples were performed to see whether or not the (temporal) changes in ARG profiles were associated with the changes in the fecal microbial population structure in response to oxytetracycline administration. The distribution of the most abundant phyla and genera in the feces over the course of the study can be seen in Fig. 7. The results showed that among the dominant group of taxa, Firmicutes exhibited low relative abundance, while the phyla Bacteroidetes and Proteobacteria exhibited increased abundances, in the medicated animals (q < 0.05, Fig. 7a, Supplementary Tables S6 and S7) on day 8 and day 21. Interestingly, the enrichment of Bacteroidetes in the feces of medicated animals was proportional to the decrease in Firmicutes abundance (Fig. 7b). This oxytetracycline-derived shift to a Bacteroidetes-dominant microbial community was also observed when the medicated animals were compared to the pretreatment animals.

**Figure 8:**
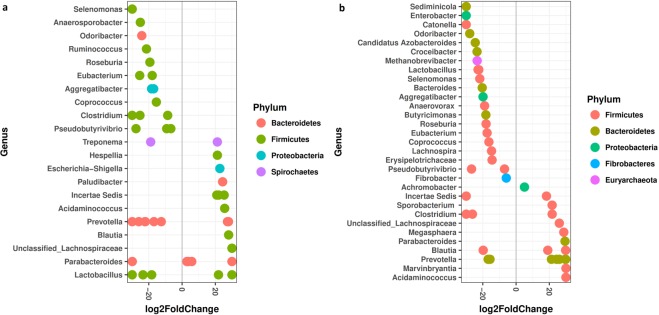
Significant (q < 0.05) log-fold changes in the abundances of bacterial species in samples from day 0 to day 8 (**a**) and day 21 (**b**). Positive log-fold change point out an increase in abundance, while negative log-fold change point out a reduction in abundance over time in the antibiotic medicated pigs compared to the control group.

Differential abundance analysis of the species-level taxonomic assignments revealed significant differences between the medicated and nonmedicated pigs microbiota. Many taxa exhibited relatively decreased abundances with antibiotic administration, most of which from the phylum Firmicutes (Fig. 8). However, the abundances of representatives of the genera *Escherichia−Shigella*, *Acidaminococcus*, *Marvinbryantia*, *Prevotella*, *Blautia*, *Parabacteroides*, *Paludibacter*, *Megasphaera*, *Clostridium*, *Sporobacterium* and *Achromobacter* and of an unclassified Lachnospiraceae were significantly enriched (q < 0.05) in the fecal microbiota of the antibiotic treated animals (Fig. 8, Supplementary Tables S8 and S9). The relative increase in the abundances of *Prevotella* spp. and *Parabacteroides* spp. was reflected by an overall increase in proportions of the phylum Bacteroidetes in the oxytetracycline-treated animals. The change in proportion of *Prevotella* was particularly notable; while this genus was among the low-abundance taxonomic groups during the pretreatment period, the abundance increased consistently over time, and *Prevotella* remained by far the most dominant taxonomic group until the end of the feeding trial.

## Discussion

One of the important questions in microbiome research relates to the extent to which production practices and environmental factors affect microbiota transmission, acquisition, and function^16^. To address this question, one approach used is experimental manipulation of gut systems to measure the impact, such as the effect of diet or antibiotic use on the microbiome^16^. In the current study, we employed shotgun metagenomics to explore the effect of in-feed oxytetracycline and its withdrawal on the dynamics of the fecal microbiota composition as well as the microbial resistome in postweaned swine over a 21-day period. Oxytetracycline is one of the most frequently employed antibiotic compounds in swine production in the European Union and the United States, with use in disease prevention as well as feed efficiency improvement^11,12,14^.

The biodiversity analysis results revealed the presence of diverse resistance genes in the fecal microbiome of the pigs, even in the absence of antibiotic pressure. In fact, ARG types, including genes encoding resistance to beta-lactams and tetracycline as well as multidrug resistance genes, were highly abundant in both medicated and nonmedicated pigs. Although there was a similarity in ARG classes detected in this study with those reported for human feces and the environmental samples^17^, the prevalent ARGs detected in this study were different from those found in human feces, river water, and sediments^17^. This finding supports the theory that specific ARGs are associated with particular environments and are not randomly distributed^18^ and that the constant selective pressure of antibiotic administration for over 50 years in swine production seems to have led to a high background level of gut resistome in swine^6^.

Oxytetracycline administration resulted in a detectable increase in the diversity and abundance of resistance genes that was even higher than the large background resistance, though the gut resistome diversity mainly recovered after two weeks of antibiotic withdrawal. Consistent with our results, Noyes *et al*.^19^ and Looft *et al*.^6^ observed an increase in the abundance and diversity of antimicrobial resistance genes in feedlot pens, where animals were administered tetracycline and ASP20 during feeding. As expected, tetracycline resistance genes were significantly enriched in the feces of the medicated animals in the current study.

Generally, efflux pumps, ribosome protection and tetracycline modification are the primary means via which bacteria are afforded resistance to tetracycline^12^. Consistent with our findings, ribosome protection seems to be the most prevalent of these mechanisms in nature^20^. The spread of the ribosomal protection proteins determinants such as *tetQ* and *tet*M throughout eubacteria via lateral gene transfer events might have been facilitated by their presence on mobile genetic elements.

Many of the resistance ribosomal protection proteins determinants such as *tetQ* and *tet*M are located on mobile genetic elements and this may have facilitated the spread of these genes throughout eubacteria via lateral gene transfer events^21^. In the current study, the *tet*Q gene, which is often associated with conjugative transposons in members of Bacteroidetes (*Prevotella, Bacteroides, Parabacteroides, Paludibacter*)^21^, represented the most dominant group of ARGs in the medicated animals, suggesting that the bacteria in the guts of medicated animals may become resistant mainly by acquisition of this gene.

As expected, oxytetracycline administration resulted in the enrichment of some tetracycline resistance genes, most likely due to a direct interaction. However, a collateral effect of antibiotic administration was observed, so that some ARGs that do not confer resistance toward oxytetracyline (e.g., *rpo*B, *ox*A, *cat*P, TEM, *mph*A, *cme*, CTX, *car*B, *gyr*A, *par*E) also exhibited increased abundance with in-feed oxytetracycline, indicating an indirect mechanism of selection. Looft *et al*.^6^ suggested that this is likely due to co-presence of some ARGs on mobile genetic elements conferring resistance to antibiotic. Accordingly, further analysis in our study revealed that a majority of these enriched ARGs have been found on mobile genetic elements such as plasmids and integrons, which carry at least two other resistance genes (data not shown). The co-occurrence of ARGs on mobile genetic element could promote spread of these genes^22^ and could further facilitate horizontal transfer of these resistance gene clusters to potential human pathogens such as *E. coli* in the swine gut or the agricultural environment^6^. Together the results showed that in-feed oxytetracycline enriched the abundance of resistance genes specific to (and beyond) the administered antibiotic in the pig fecal microbiome.

Based on the analyses of the microbiota, we conclude that the fecal microbial diversity increases over time and shifted to an adult-type microbiota, which is consistent with previous studies made in pig^23–26^. Overall, Firmicutes and Bacteroidetes phyla were the predominate taxa in the fecal microbiota of the pigs, accounting for more than 90% of the bacterial population during the post weaning period^24,27,28^. Analysis also revealed that the therapeutic dose of oxytetracycline caused a reduction in overall species richness and diversity in the medicated animals and that the reduction lasted even after antibiotic administration was discontinued. Although the reduction was not statistically significant at the community level, the antibiotic treatment resulted in significant and enduring changes at the species level, indicating that a particular group of the microbial communities could confer greater resistance to perturbance induced by antibiotic than other gut microbiota members, which could be due to the specific effect the antibiotic^29^.

In this study, the most notable change in bacterial abundance was the increase in the abundances of Bacteroidetes and Proteobacteria during the first 7 days of oxytetracycline exposure, which was mainly observed as increased *Prevotella*, *Parabacteroides*, *Paludibacter* (Bacteroidetes) and *Escherichia* (Proteobacteria) abundances. Similar to our findings, ASP250 administration for three weeks has been shown to cause detectable divergence in the swine gut microbiota, including an increase in Proteobacteria abundance, which was correlated with increased *Escherichia* spp. abundance^6^. However, when amoxicillin and the β-lactamase inhibitor clavulanic acid were applied together, in the feed and via intramuscular injection, decreased *E. coli* abundance was observed in pigs^30^. *Escherichia* has been found to encode various ARGs, such as resistance genes for beta-lactams (*cfx*A3) and tetracycline (*tet*Q), genes for multidrug resistance (*acr*A, *mdt*H, *mdt*L and *mdt*O), and other genes (dimethyladenosine transferase)^31^. The phylum Bacteroidetes has been found to decrease in pigs fed tylosin^32^ and ASP250^6^, while carbadox administration has been reported to increase the abundance of this phylum during the early phase of administration^4^. An increase in the ratio of Bacteroidetes to Firmicutes proportion has been recently linked to increased short-chain fatty acid (SCFA) production in mice in response to fructo-oligosaccharide administration^33^. However, other studies have also highlighted possible negative impacts of enriched Bacteroidetes populations in the gut^34,35^. In terms of the gut resistome, the observed increase in the abundances of *Prevotella*, *Parabacteroides*, and *Paludibacter* in the medicated animals in the present study might be due to the potential of these taxa to carry ARGs that may be transferred to other susceptible bacteria in the densely populated microbial environment like the swine gut^36,37^.

Interestingly, previous studies have clearly highlighted the occurrence of tetracycline resistance genes (mainly *tet*Q) in taxa from *Escherichia*, *Parabacteroides* and *Prevotella*^21,31,37,38^. Recently, the relative abundances of *Prevotella*, *Paludibacter*, and *Parabacteroides* have been reported to be significantly correlated with the abundances of aminoglycoside, beta-lactam, MLS, sulfonamide, and tetracycline resistance genes and the abundances of transposases^39^. *Blautia*, *Acidaminococcus* and *Megasphaera* from Firmicutes were also found to be significantly enriched in the feces of the medicated swine. *Blautia* has been reported to harbor tetracycline resistance genes (*tet*Q, *tet*O, *tet*32, *tet*M) and a MLS resistance gene (*erm*B)^31,38^. Similarly, *Acidaminococcus* and *Megasphaera* have also been reported to carry tetracycline resistance genes (*tet*O, *tet*W)^40,41^. Overall, different patterns of shifts in microbial populations have been reported when different antibiotics were administered to pigs^4,6,28,42^, indicating that the effects of antibiotics on some microbial members are specific to the antibiotic being administered and depend on the varying collateral effects of different antibiotics.

In this study, the experimental design featured environmental controls such as host genetic control, no application of antibiotics to the sows or pigs to prior the experiment, and identical diet except for the inclusion of oxytetracycline for one treatment group. However, a limitation of the present study is that resistome profiling of the feeding trial facility environment as well as the feed samples in the pre and post weaning phase was not considered. The lack of this information may have impacted the accuracy of our findings to some extent. Despite this limitation, this study represents the first report on using shotgun metagenomics for studying dynamics of the gut microbiome and antibiotic resistome alterations in swine.

Further research is recommended to look beyond metagenomics-based resistome profiling and at effects on (AR) gene expression and even on the proteome and metabolome level. Additionally, given the widespread distribution of phages in the gut environment, the role of phages in the acquisition and spread of ARGs should be considered in future studies. Despite the recent observation that ARGs are rarely encoded in phage genomes^43^, the bacterium-phage interaction and subsequent (antibiotic resistance) gene transfer in the gut environment has not been fully investigated.

## Conclusions

In this study, the collateral effects of in-feed oxytetracycline administration at therapeutic dose on the pig fecal antibiotic resistome are observed. Even a short-term administration of oxytetracycline increased the abundance and diversity of ARGs, including those conferring resistance to antibiotics that were not administered, and increased the abundance of Proteobacteria, including *E. coli* population, a potential human pathogen. Although the effect of the therapeutic application on ARGs diminished over time, some ARGs remained significantly more abundant (q < 0.05) in medicated pigs than in the control group two weeks after the withdrawal of antibiotic administration.

## Materials and Methods

### Statement

The animal experiments were conducted under a protocol approved by the office of the Lower Austrian Region Government, Group of Agriculture and Forestry, Department of Agricultural Law (approval codes LF1-TVG-39/038-2016). The trial was carried out at the Center of Animal Nutrition (Tulln, Austria). All experiments and methods were conducted according to relevant guidelines and regulations.

### Animals and experimental design

Sixteen freshly weaned pigs (sow: Landrace × Large White, boar: Pietrain) that were ∼28 days old were selected for this study. Upon arrival the animals were individually housed and maintained in similar climatically controlled rooms. After four days of adaptation with *ad libitum* access to a standard starter diet (Table 1), the pigs were blocked (row-column design^44^) by sex (2) and ancestry (4), and within each block, the animals were randomly allocated to one of two treatments (n = 8 pigs/treatment): 1) standard starter diet for 21 days (control group) or 2) antibiotic-supplemented diet (10 g oxytetracycline Agrar-Service/100 KBW/day, corresponding to 40 mg oxytetracycline hydrochloride/KBW/day) for 7 days (recommended therapeutic dosage by the manufacturer). The treatment was followed by 14 days of standard starter diet (antibiotic group). For the duration of the study, the pigs were allowed *ad libitum* access to water and feed and all dietary treatments were equally represented in each room to remove any variation due to environmental factors.

**Table 1:** Composition of the pig starter diet.

Ingredient	%	Nutrient	g/kg (as fed basis)
Barley	29.7	Metabolize energy MJ/kg	13.2
Wheat	10.0	Crude protein	165
Corn	10.8	Ash	50
Reapeseed oil	0.45	Crude fiber	38
Full fat soya	14.13	Crude fat	69
Wheat pressure cooked	9.90	Lysine	14.0
Potato protein	5.04	Ileal digestible lysine	12.5
Whey powder	2.97	Methionine	5.5
Vinasses	1.85	Ileal digestible methionine	4.9
Dextrose	5.04	Threonine	9.1
Palm kernel	1.98	Ileal digestible threonine	7.8
Lactose	2.97	Tryptophan	2.6
Lignocellulose	0.90	Ileal digestible tryptophan	2.1
Mono calcium phosphate	1.35	Valin	9.4
Calcium carbonate	0.63	Ileal digestible valine	6.6
Sodium chloride	0.45	Ca	6.4
Magnesium phosphate	0.18	P	6.2
Trace min.–vit. Premix (vitamin A 16000 IU, vitamin D3 2000 IU, vitamin E 150 IU, vitamin K3 0.4 mg, vitamin B1 2.8 mg, vitamin B2 8.2 mg, D-pantothenic acid – 20 mg, biotin 50 μg, vitamin B12 50 μg, folic acid 1.05 mg, vitamin B6 5 mg, cholinechloride 500 mg, nicotinic acid 60 mg, Fe 124 mg, Zn 121 mg, Mn 80 mg, Co 124 mg, I – 3.1 mg, Se 0.45 mg, phytase 250 FTU and antioxidants 100 mg).	0.36	Na	2.3
		Mg	1.7
DL-Methionine	0.27		
L-lysine	0.70		
L-Tryptophan	0.06		
L-Threonine	0.26		

### Fecal sampling, DNA extraction, library preparation and sequencing

Fecal materials were obtained from the individual pig by rectal stimulation on days 0 (before oxytetracycline treatment), 8 (after the oxytetracycline treatment), and 21 (two weeks after the withdrawal of oxytetracycline), and stored in sterile containers at –20 °C until processed. The total DNA was extracted from the fecal samples by the QIAamp PowerFecal Kit (Qiagen, Crawley, West Sussex, UK) following the manufacturer’s instructions with some modifications recommended by Hart *et al*.^45^. The final DNA were eluted in 100 μL of 10 mM Tris buffer (pH 8) after being incubated for 5 min for maximum elution efficiency. A Qubit fluorometer (Qubit 3, Invitrogen) was used to determine the total DNA concentration, and purity was assessed via the 260/280 and 260/230 absorbance ratios using a spectrophotometre (NanoDrop® ND-1000). The samples were sent for DNA sequencing to the Teagasc Food Research Centre, Ireland. Paired-end sequencing libraries were prepared from the extracted DNA using the Illumina Nextera XT Library Preparation Kit (Illumina Inc., San Diego, CA) followed by sequencing on the Illumina NextSeq 500 platform using high-output chemistry (2 × 150 bp) according to the manufacturer’s instructions.

### Sequence analysis

All bioinformatics and statistical analyses of the metagenome datasets were conducted with custom Bash, R, and Perl scripts using the existing softwares and algorithms. (see below).

### Quality filtering

Quality filtering of the metagenome datasets was carried out in several steps to remove sequencing adapters (cutadapt v. 1.12^46^), low-quality sequences with quality scores < 33 (fastx toolkit v. 0.0.14), reads mapped to the host genome (pig, NCBI accession no. NC 010443) (DeconSeq v. 0.4.3^47^), and finally to remove any sequences mapped to the PhiX174 genome (NCBI accession no. NC 001422) (DeconSeq v. 0.4.3^47^).

### Resistome annotation and comparison

To quantify the abundances of ARGs, 42 quality-filtered metagenomes were used for similarity searches against the hand-curated antimicrobial resistance database MEGARes^48^ by using USEARCH (v10)^49^. Containing the sequences of approximately 4,000 ARGs, the MEGARes database is based on a nonredundant compilation of sequences contained in ResFinder (November 2015), ARG-ANNOT (November 2015), the Comprehensive Antibiotic Resistance Database (CARD, v1.0.7), and the National Center for Biotechnology Information (NCBI) Lahey Clinic beta-lactamase archive (December 2015)^48^.

High confidence matches to the sequence in MEGARes database were obtained by considering the entire coverage of the query reads against ARGs genes with a identity threshold of 90% (parameters were set as “-usearch-global -id 0.9, maxaccepts 1, threads 50”), as suggested elsewhere^17^. For each antibiotic resistance determinant (ARD), the total number of aligned reads was counted followed by normalization to the length of the respective gene, in order to remove possible sequence length variations bias^17^. Further, the length-normalized counts were normalized to the bacterial *16S r*RNA sequences number (obtained by employing Metaxa2^50^) divided by the average length of the *16S* gene to yield an approximation of the ARGs number per bacterial *16S r*RNA^17^ (Equation 1).

1 $$Abundance=\sum _{1}^{n}\frac{{N}_{{\rm{AMR}} \mbox{-} {\rm{like}}\mathrm{sequence}}/{L}_{AMRreferencesequence}}{{N}_{16Ssequence}/{L}_{16Ssequence}}$$

### Taxonomic affiliation

The taxonomic compositions of the metagenome datasets were identified by extracting the bacterial *16S r*RNA sequences with Metaxa2 version 2.0 using the default options^50,51^. Genus assignment of the extracted sequences was carried out using the Metaxa 2 curated database taking to the account the reliability score (>80) as well as the similarity threshold (>90% identity with the reference 16*S r*RNA sequence) and reported as relative abundance based on the total number of *16S r*RNA counts in each metagenome sample.

### Statistical analysis

Ordination and log-fold changes in abundance were calculated in R (version 3.3.0). Ordination was performed with log-transformed normalized reads on 2 dimensions with the “phyloseq’s” ordinate function using Non-metric multidimensional scaling (NMDS) analysis^52^. On the completed ordination plots, separation between groups was tested with PerMANOVA^53^. Log-fold changes in abundance (of taxa and ARGs) between groups was determined by a negative binomial generalized linear model using DESeq2 version 1.17.10^54^ in R, considering random differences between the treatment groups at first sampling as covariates term in the model. Accordingly, treatment and sampling day were included as fixed factors, while blocks were considered as confounder variables (random factors) in the analysis. These main factors along with their interactions were also taken into the account to investigate richness (the number of unique taxa or ARGs) and Shannon diversity (the number and relative abundance of unique taxa or ARGs) in each sample using the lme4 package in R^55^. Statistical significance for differential abundance analysis was considered at FDR-corrected P ≤ 0.05 (where applicable) and shown as the q value. In all statistical analysis, the individual animal/pen was considered the experimental unit.

## Supplementary information

Supplementary file S1

## Data Availability

The data are deposited in the NCBI Short Read Archive under BioSamples SAMN09209536- SAMN09209575, which are affiliated with BioProject PRJNA471402.
